# Clinical Characteristics and Prognoses of Patients With Systemic Lupus Erythematosus Hospitalized for Pulmonary Infections

**DOI:** 10.3389/fmed.2021.732681

**Published:** 2021-09-29

**Authors:** Yanli Yang, Hui Jiang, Chuhan Wang, Nan Jiang, Chanyuan Wu, Shangzhu Zhang, Wei Jiang, Jinmin Peng, Li Weng, Jiuliang Zhao, Qian Wang, Mengtao Li, Bin Du, Yan Zhao, Xiaofeng Zeng

**Affiliations:** ^1^Department of Rheumatology and Clinical Immunology, National Clinical Research Center for Dermatologic and Immunologic Diseases (NCRC-DID), Peking Union Medical College, Peking Union Medical College Hospital, Chinese Academy of Medical Sciences, Beijing, China; ^2^Shanxi Bethune Hospital, Tongji Shanxi Hospital, Third Hospital of Shanxi Medical University, Shanxi Academy of Medical Sciences, Taiyuan, China; ^3^Department of Medicine Intensive Care Unit, National Clinical Research Center for Dermatologic and Immunologic Diseases (NCRC-DID), Peking Union Medical College, Peking Union Medical College Hospital, Chinese Academy of Medical Sciences, Beijing, China

**Keywords:** systemic lupus erythematosus, pulmonary infection, mortality, risk factor, cardiopulmonary involvement, opportunistic infection

## Abstract

**Objective:** To identify factors associated with mortality in SLE patients who were hospitalized for pulmonary infections (PIs).

**Methods:** This single-center retrospective study analyzed the characteristics and risk factors for mortality in 95 SLE patients hospitalized for PIs.

**Results:** Ninety-five SLE patients had 97 episodes of hospitalization for PIs, and 33 of these episodes (34.02%) led to death. Death from PI was associated with a higher neutrophil count (6.30 vs. 4.201 × 10^9^/L, *p* < 0.01), immunoglobulin G (6.20 vs. 9.82 g/L, *p* = 0.01), serum creatinine (126.00 vs. 73.00 μmol/L, *p* = 0.01), proteinuria (2.99 vs. 0.54 g/day, *p* < 0.01), cardiopulmonary involvement (57.58 vs. 34.38%, *p* < 0.05), SLE disease activity index (SLEDAI; 11.00 vs. 6.00, *p* < 0.05), and opportunistic infections (78.79 vs. 45.31%, *p* < 0.05). Demographic characteristics, antibody/complements, bacterial infection, and primary treatment before infection (including corticosteroid and immunosuppressants) had no effect. Multivariate analysis indicated cardiopulmonary involvement (HR: 2.077; 95%CI: 1.022–4.220; *p* = 0.043) and opportunistic infection (HR: 2.572; 95%CI: 1.104–5.993; *p* = 0.029) were independent risk factors for mortality. High-dose steroid pulse therapy (HR: 0.982; 95%CI: 0.410–2.350; *p* = 0.982) and first-line immunosuppressant therapy (HR: 1.635; 95%CI: 0.755–3.542, *p* = 0.212) had no effect on mortality.

**Conclusion:** Cardiopulmonary involvement and opportunistic infection were independent risk factors for mortality for SLE patients hospitalized for PIs. Use of high-dose pulse steroids and or immunosuppressants before hospitalization had no significant effects.

## Introduction

Systemic lupus erythematosus (SLE) is a chronic systemic autoimmune disease characterized by a broad range of clinical manifestations (1). The most common symptoms are malar rash, inflammation of peripheral joints, and fatigue (often due to anemia). Because of improvements in the early diagnosis and treatment of SLE during recent decades, the 5-year survival rate of patients has significantly improved (2). With appropriate treatment, many patients currently diagnosed with SLE, even those with multiple morbidities, can have nearly normal lifespans (2).

However, a meta-analysis reported a 3-fold increase in all-cause mortality for patients with SLE compared to the general population (3). In Great Britain, patients with SLE had a 1.8-fold increased mortality rate compared with the general population (4). Several recent studies focused on the risk factors for infection in patients with SLE (5–7). Infections in general, and especially pulmonary infections (PIs), are a major cause of hospitalization and mortality in patients with SLE. A recent study reported that 31.1% of deaths in SLE patients were caused by infections, most commonly pneumonia due to Gram-negative bacilli (8). The above-mentioned meta-analysis (3) showed that the standardized mortality risk due to infections in SLE patients was 5-times greater than in the general population (3). Other factors, including older age at disease onset, autoimmune hemolytic anemia, thrombocytopenia, and pulmonary arterial hypertension, are also associated with poor outcomes (8).

A previous study with a small sample size examined the prognosis of SLE patients who had PIs (9). The present study of SLE patients with PIs examined a larger sample of patients and used a more rigorous statistical analysis to identify factors independently associated with short-term mortality in SLE patients who were hospitalized for PIs.

## Materials and Methods

### Patient Characteristics

This single-center retrospective study examined 95 SLE patients hospitalized for PIs in the Department of Rheumatology and Immunology or the Internal Medicine Intensive Care Unit (MICU) of Peking Union Medical College Hospital from January 2015 to December 2019. All patients met the 1997 SLE classification criteria of the American College of Rheumatology (10). Patients with tumors were excluded. This research was approved by the local ethics committee (Approval #: JS-2038).

### Definitions and Etiology of Infections

Bacterial infections were determined mainly by culture or microscopic examination. Fungal infections were determined mainly according to the criteria of the 2008 consensus group of the European Organization for Research and Treatment of Cancer/Invasive Fungal Infections Cooperative Group and the National Institute of Allergy and Infectious Diseases Mycoses Study Group (11). Viral infections were determined by detection of replicated viral nucleic acids using the polymerase chain reaction (PCR) and confirmed by analysis of clinical features (fever, pharyngitis, arthralgia, and rash), specific chest CT changes (ground-glass changes and fine-grid changes), and laboratory tests (virus-specific immunoglobulin M [IgM]). Tuberculosis was diagnosed according to positive *Mycobacterium tuberculosis* in culture or microscopic examination. Episodes without specific pathogenic findings were analyzed in terms of clinical symptoms, high-resolution chest computed tomography (HRCT), laboratory determinations of white blood cell count (WBC), erythrocyte sedimentation rate (ESR), procalcitonin (PCT), interferon-gamma release assay or tuberculin skin test, and response to antibiotic therapy. Opportunistic infection was defined by an episode of at least one infection by a fungus, virus, or *M. tuberculosis*.

### Data Collection

Data collected at the time of hospital admission included demographic characteristics, clinical characteristics, laboratory indicators, SLE activity index (SLEDAI) (12), complications, and therapies. Records of the involvement of different Organs [skin involvement, lupus nephritis, hematologic system involvement, serositis, arthritis, myositis, and cardiopulmonary involvement (cardiomyopathy, alveolar hemorrhage and pulmonary hypertension)] were also recorded. Laboratory indexes included leukocytes (related to infection), 24-h urine protein, complements, anti-dsDNA (related to SLE activity), and other systemic biochemical indexes. Use and dosage of glucocorticoids, steroid pulse therapy, and immunosuppressants during the 3 months before hospitalization were recorded. Glucocorticoid dosage was converted to prednisolone dosage according to the following formula: 1 mg prednisolone = 0.8 mg methylprednisolone = 0.15 mg dexamethasone. The duration of hospitalization was recorded, and the longest hospital stay was 120 days. The end point was death, and survival episodes after leaving the hospital were censored.

### Statistical Analysis

All data were analyzed using SPSS version 25.0. Continuous variables were expressed as means ± standard deviations (SDs) or medians (interquartile ranges [IQRs]). Comparison between groups was conducted using Student's *t*-test or the Wilcoxon-Mann-Whitney test. Categorical variables were presented as frequencies and percentages, and the chi-square test or Fisher's exact test was used for comparisons as appropriate. The Kaplan-Meier method was used to compare mortality of different groups during hospitalization. Single-factor items with statistical difference were analyzed by Cox multivariate regression analysis to identify independent and significant risk factors. A *P* < 0.05 indicated statistical significance.

## Results

### Demographics and Organ Involvement

We investigated 95 SLE patients with PIs. There were 97 episodes of PI, with three episodes in one patient. The causes were pathogens of unknown etiology, *Nocardia* sp., and oxacillin-resistant *Staphylococcus aureus*, or *Cytomegalovirus*. Thirty-three episodes (35%) died and 64 episodes (65%) survived (Table 1). The two groups had no significant differences in age (37.0 ± 14.8 vs. 38.8 ± 14.1 years, *p* > 0.05), percentage of males (21.21 vs. 10.94%, *p* > 0.05), or median duration of SLE (60.00 vs. 24.00 months, *p* > 0.05). The survival group had a marginally longer time of hospitalization (*p* = 0.045). There were 19 episodes combined with cardiopulmonary manifestations in the deceased group (three with pulmonary hypertension, three with alveolar hemorrhage, and 13 with cardiomyopathy), while 22 episodes in the survival group (five with pulmonary hypertension, four with alveolar hemorrhage, and 13 with cardiomyopathy). Thus, cardiopulmonary involvement was more common in the deceased group (57.58 vs. 34.38%, *p* = 0.033). There were no significant differences in the other clinical manifestations (all *p* > 0.05). Notably, the two groups had no significant differences in the prevalence of type 2 diabetes (21.21 vs. 10.94%, *p* > 0.05) or bloodstream infections (63.64 vs. 43.75%, *p* > 0.05).

**Table 1:** Demographic characteristics and organ involvement in the two groups^*^.

**Variable**	**Deceased group (*n* = 33)**	**Survival group (*n* = 64)**	***P* value**
Age, years, mean ± SD	37.01 ± 4.8	38.81 ± 4.1	0.830
Male, *n* (%)	7 (21.21)	7(10.94)	0.224
Disease duration, months, median (IQR)	60.00 (6.00–120.00)	24.00 (4.25–96.00)	0.396
Hospitalization time, median (IQR)	21.00 (7.00–36.50)	26.00 (17.30–38.80)	***0.045***
Lupus nephritis, *n* (%)	28 (84.85)	42 (65.63)	0.057
Skin and mucosa lesions, *n* (%)	2 (6.06)	8 (12.50)	0.487
Hematologic disorder, *n* (%)	24 (72.73)	41 (64.06)	0.496
Arthritis, *n* (%)	1 (3.03)	1 (1.56)	1.000
Myositis, *n* (%)	2 (6.06)	3 (4.69)	1.000
Digestive disorder, *n* (%)	3 (9.09)	14 (21.88)	0.161
Serositis, *n* (%)	9 (27.27)	11 (17.19)	0.293
Neuropsychiatric disorder, *n* (%)	12 (36.36)	13 (20.31)	0.140
Hemolytic anemia, *n* (%)	3 (9.09)	2 (3.13)	0.333
Cardiopulmonary disorders, *n* (%)	19 (57.58)	22 (34.38)	***0.033***
Type 2 diabetes mellitus, *n* (%)	7 (21.21)	7 (10.94)	0.224
Bloodstream infections, *n* (%)	21 (63.64)	28 (43.75)	0.087

* *Age is given as mean ± SD, gender as males/females (percent males), and disease duration as median (IQR). All other data are given as n (%). Bold values means p value < 0.05 compared to the two groups*.

### Laboratory Data and Use of Corticosteroids and Immunosuppressants

We also compared the laboratory data and treatments in the two groups (Table 2). The two groups had no statistically significant differences in lymphocyte count, complements, or anti-dsDNA positivity (all *p* > 0.05). However, the deceased group had significantly higher absolute neutrophil count (ANC; 6.30 vs. 4.20 × 10^9^/L, *p* < 0.01), 24-h urine protein (24 h-UPro; 2.99 vs. 0.54 g, *p* < 0.01), and serum creatinine (SCr; 126.00 vs. 73.00 μmol/L, *p* = 0.01), and significantly lower levels of immunoglobulin G (IgG; 6.20 vs. 9.82 g/L, *p* = 0.01) and serum albumin (27.00 vs. 29.00 g/L, *p* = 0.009). The deceased group also had a higher median SLEDAI (11.00 vs. 6.00, *p* < 0.05).

**Table 2:** Laboratory data and medications in the two groups^*^.

	**Deceased group (*n* = 33)**	**Survival group (*n* = 64)**	***P* value**
**Laboratory tests at admission**			
Neutrophil count (×10^9^/L), median (IQR)	6.30 (4.13–8.71)	4.20 (2.50–6.80)	***0.009***
Lymphocyte count (×10^9^/L), median (IQR)	0.36 (0.18–0.59)	0.45 (0.27–0.93)	0.077
24-hour urine protein (g), median (IQR)	2.99 (1.03–5.10)	0.54 (0.29–1.66)	*** < 0.001***
Albumin (g/L), median (IQR)	27.00 (22.50–29.00)	29.00 (25.25–34.00)	***0.009***
Serum creatinine (μmol/L), median IQR)	126.00 (79.00–199.00)	73.00 (50.25–151.50)	***0.010***
Immunoglobulin G (g/L), median (IQR)	6.20 (4.32–9.70)	9.82 (5.70–16.00)	***0.010***
Complement 3 (g/L), median (IQR)	0.59 (0.38–0.74)	0.66 (0.39–0.91)	0.444
Complement 4 (g/L), median (IQR)	0.12 (0.69–0.16)	0.13 (0.06–0.19)	0.747
SLEDAI, median (IQR)	11.00 (6.00–16.00)	6.00 (2.25–10.75)	***0.003***
Anti-dsDNA antibody positivity, *n* (%)	8 (24.24)	21 (32.81)	0.483
**Infection status**			
Bacterial infection, *n* (%)	21 (63.64)	33 (51.56)	0.257
Opportunistic infection, *n* (%)	26 (78.79)	29 (45.31)	***0.002***
Mixed infection, *n* (%)	15 (45.45)	15 (23.44)	***0.026***
Drug-resistant bacteria, *n* (%)	14 (42.42)	17 (26.56)	0.112
**Primary therapy before hospitalization**			
Glucocorticoid dose (mg/day), median (IQR)	37.50 (7.50–57.50)	25.00 (0.00–39.50)	0.081
Glucocorticoid duration (months), median (IQR)	30.00 (5.25–93.00)	14.00 (1.50–72.00)	0.211
Steroid pulse therapy, *n* (%)	9 (27.27)	11 (17.19)	0.293
Tripterygium glycosides, *n* (%)	1 (3.03)	2 (3.13)	1.000
Any immunosuppressant, *n* (%)	24 (72.72)	40 (62.50)	0.370
MMF, *n* (%)	5 (15.15)	7 (10.94)	0.535
MTX, *n* (%)	0 (0.00)	1 (1.56)	1.000
CsA/FK506, *n* (%)	3 (9.09)	7 (10.94)	1.000
LEF, *n* (%)	1 (3.03)	0 (0.00)	0.340
Multi-target treatment, *n* (%)	4 (12.12)	10 (15.63)	0.340

* *Data are given as median (IQR) or n (%). MMF, mycophenolate mofetil; MTX, methotrexate; CsA/FK506, cyclosporin A/tacrolimus; LEF, leflunomide; TG, tripterygium glycoside. Bold values means p value < 0.05 compared to the two groups*.

Analysis of infections indicated 76 episodes with positive culture or microscopic findings (47 in the survival group, 29 in the deceased group), but failure in pathogen identification in the other 21 episodes (17 in the survival group, four in the deceased group). The two groups were similar in terms of bacterial infections (63.64 vs. 51.56%, *p* > 0.05), but the deceased group had more opportunistic infections involving fungi, viruses, and *M. tuberculosis* (78.79 vs. 45.31%, *p* < 0.05). The deceased group also had a higher prevalence of mixed infections of bacteria and opportunistic infections (45.45 vs. 23.44%, *p* = 0.026). Drug-resistant bacteria were reported in 31 episodes including pan-drug resistant *Acinetobacter baumannii* (XDRAB), carbapenem-resistant *Enterobacteriaceae* (CRE), carbapenem-resistant *Enterobacteriaceae* (MRSA), methicillin-sensitive *Staphylococcus Aureus* (MSSA), extended-spectrum β-lactamases positive (ESBL+) bacteria, and others (17 in the survival group, 14 in the deceased group, *p* > 0.05) (Table 2).

Analysis of corticosteroid therapy before hospitalization (Table 2) indicated the two groups had no significant differences in therapy duration before PI, average daily dosage of glucocorticoid during the preceding 3 months, and use of steroid pulse therapy (all p > 0.05). Twenty-four episodes in the deceased group (72.72%) and 40 episodes in the survival group (62.50%) received immunosuppressive therapy, and the two groups had were no significant differences in pre-hospitalization therapies (all *p* > 0.05).

### Risk Factors for Death

We performed a multivariate Cox regression analysis to identify factors associated with mortality during hospitalization using single items that were statistically different and with appropriate adjustment (Table 3). Thus, ANC, 24h-UPro, SCr, IgG, albumin, SLEDAI, opportunistic infections, and cardiopulmonary involvement were analyzed in the multivariate analysis. The results indicated that cardiopulmonary involvement (HR: 2.077; 95% CI: 1.022–4.220; *p* = 0.043) and opportunistic infection (HR: 2.572; 95% CI: 1.104–5.993; *p* = 0.029) were independent risk factor for mortality in these patients.

**Table 3:** Multivariate Cox regression analysis of factors associated with 120-day mortality.

**Variable**	**HR**	**95% CI**	***P***
Elevated neutrophils^*^	1.688	0.671–4.248	0.266
Elevated 24 h UPro^*^	1.359	0.349–5.297	0.659
Serum albumin	1.002	0.936–1.073	0.952
Serum IgG	1.463	0.663–3.231	0.346
Serum creatinine	0.999	0.997–1.001	0.399
SLEDAI ≥ 5^*^	1.793	0.550–5.843	0.333
Opportunistic infection	2.572	1.104–5.993	***0.029***
Cardiopulmonary involvement	2.077	1.022–4.220	***0.043***

* *Elevated neutrophils, neutrophils over 7.51 × 10^9^/L, Elevated 24hUPro, urine protein over 0.5g/24h, SLEDAI over 5 means activity of SLE. Bold values means p value < 0.05 compared to the two groups*.

### Survival Analysis

**Figure 1:**
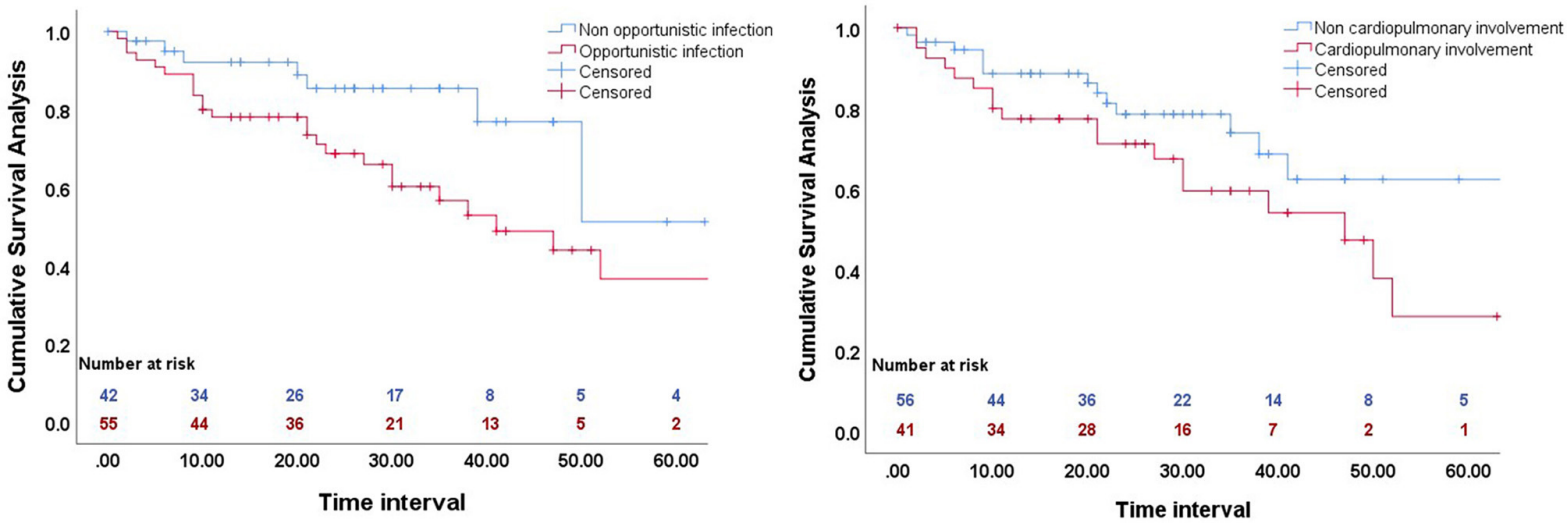
Kaplan-Meier analysis of the effect of opportunistic infection (left), and cardiopulmonary involvement (right) on cumulative overall survival.

We performed Kaplan–Meier analysis to determine the effect of opportunistic infection and cardiopulmonary involvement on cumulative overall survival (Figure 1). The results indicated worse survival in patients with opportunistic infections (*p* = 0.036), but cardiopulmonary involvement had no significant effect (*p* = 0.064).

## Discussion

PI is a leading cause of death in patients with SLE, and it is therefore important for clinical practitioners to evaluate the severity and prognosis of SLE patients who are admitted with PIs. Fei et al. (13) reported that the death rates of SLE patients due to infections in Peking Union Medical College Hospital increased from 25.7% during 1986–1990 to 60.3% during 2006–2011 and that 65% of those who died from infections had PIs. However, recent research mainly focused on the risk factors for infection in SLE patients, and devoted little attention to the risk factors for PI-related death (9). There is therefore only limited understanding of the factors associated with disease progression in SLE patients with PIs. The current study examined factors associated with survival in SLE patients with PIs by retrospective analysis of the clinical data of 95 SLE patients hospitalized for PIs at Peking Union Medical College Hospital from January 2015 to December 2019. The results indicated that cardiopulmonary involvement and opportunistic infection were independent risk factors for mortality, but receipt of high-dose pulse steroid therapy and immunosuppressive therapy before hospitalization had no effect on mortality.

During the time of hospitalization, 33 of our patients (34%) died. The deceased group and survival group had statistically significant differences in ANC, IgG, SCr, serum albumin, proteinuria, cardiopulmonary involvement, SLEDAI score, and prevalence of opportunistic infection (all *p* < 0.05). Multivariate Cox regression analysis showed that cardiopulmonary involvement and opportunistic infection were independent risk factors for mortality. However, use of high-dose steroid pulse therapy or a first-line immunosuppressant prior to hospitalization had no effect on survival.

Previous studies showed that male gender, fungal infection, elevated 24-h urine protein, elevated absolute lymphocyte count, and low C3 level increased the risk for death from PI in patients with SLE (9, 14–18). Our results confirmed that cardiopulmonary involvement and opportunistic infection were risk factors in these patients. Mu et al. (8) reported that the risk factors for poor prognosis of SLE patients in China were advanced age at disease onset, infection, autoimmune hemolysis, thrombocytopenia, and pulmonary hypertension. Bacteria are the most common infectious pathogens in our SLE patients, followed by fungi, viruses, and *M. tuberculosis*. Most studies demonstrated that corticosteroid therapy increased the risk of infection, and that hydroxychloroquine sulfate protected against infection (7). However, reports on the influence of immunosuppressants on infections in patients with SLE are inconsistent. Studies of the effect of gender on mortality from SLE-associated PI have also been inconsistent. Wu et al. (19) reported that males with SLE had an increased risk for death from PI, but Mu et al. (8) believed that the gender difference may be due to the significantly reduced participation of males and the shorter life expectancy of men in general. The current study showed no statistical effect of gender on mortality. In addition, the present study also indicated that cardiopulmonary involvement (myocarditis, alveolar hemorrhage, and pulmonary hypertension) was an independent risk factor for death. Patients with respiratory and circulatory system involvement mostly had acute and critical progression, and this contributed to their poor prognosis.

Contrary to the results of Lu et al. (9), our results showed that opportunistic infections from fungi, viruses, and *M. tuberculosis* were more common in the deceased group, but the two groups had no significant difference in the prevalence of bacterial infections. A retrospective study performed in northern China reported that invasive fungal infections without timely treatment may lead to a mortality rate as high as 30–80% in patients with connective tissue diseases (19). Moreover, long-term use of multiple antibiotics increased the risk of secondary fungal infections, and these tended to be asymptomatic, rapidly progressive, and associated with poor prognosis. This may have been the reason for the statistical difference between these two groups. On the other hand, the discrepancies between our results and these previous results (19) may be attributable to the use of different methods for detection of pathogenic bacteria. In particular, the current study sampled fungal and viral DNA in most deceased patients using fibrobronchoscopic alveolar lavage fluid, and this might have contributed to the statistically significant differences between the survival and deceased groups.

Previous reports that examined the effects of steroid and immunosuppressant therapy on the prognosis of patients with SLE and infections were inconsistent, although most showed that steroid use increased the risk of infection. For example, a recent study of 803 patients from Hong Kong who were followed for an average of 10 years found that use of prednisone at a dose exceeding 0.6 mg/kg/day for at least 4 weeks was associated with a more than 14-fold increased risk of all-cause mortality (20). However, other studies reported that methyl-prednisolone pulse therapy was not associated with organ damage in SLE patients (21, 22). In contrast, other research showed that the use of immunosuppressive drugs did not increase the risk of severe infection and death in SLE patients (23). Similarly, for SLE patients with bacteremia, use of an immunosuppressive drug or a corticosteroid was not associated with increased mortality (24).

Our research showed that the duration of steroid use, average daily steroid dose during the 3 months before PI, and steroid pulse therapy did not increase mortality. The survival and deceased groups also had no significant difference in use of immunosuppressants. The use of a steroid or an immunosuppressant certainly weakens resistance to pathogenic bacteria, and the natural course of SLE often leads to the involvement of major organs. In addition, SLE may be associated with immune defects, such as complement alterations, functional decline of phagocytes, and impaired cytokine production (7), and these alterations lead to an increased susceptibility to complicated infections. Active and appropriate treatment with corticosteroids and/or immunosuppressants can therefore prevent disease progression and improve prognosis in patients with SLE. Importantly, although steroid pulse therapy can increase the risk of infection, it does not increase the risk of mortality.

This study has several limitations. First, we conducted this study at a single center. Second, we only evaluated SLE patients with PIs who died soon after diagnosis. Further prospective studies are therefore needed to assess prognostic factors that affect long-term survival. In addition, we only retrospectively examined a small number of SLE patients with PIs. A prospective randomized controlled study of a large number of such patients is needed for confirmation. A final limitation is that complete data regarding changes of different indicators during hospitalization were not available for all inpatients, and this made it difficult the track the effects of therapy.

Our results indicated that cardiopulmonary involvement and opportunistic infection were independent risk factors for short-term mortality in SLE patients with PIs. In terms of clinical management, timely and effective anti-infection treatment is required for such patients, especially those with fungal and viral infections. At the same time, treatment of active SLE should not be neglected. For patients requiring high-dose steroid pulse therapy, treatment for the primary disease of SLE must be strengthened to prevent progression, because this will likely improve patient prognosis.

## Data Availability Statement

The original contributions presented in the study are included in the article/supplementary material, further inquiries can be directed to the corresponding authors.

## Ethics Statement

The studies involving human participants were reviewed and approved by the Ethics Committee of Peking Union Medical College Hospital and the patients provided written informed consent. Written informed consent to participate in this study was provided by the participants' legal guardian/next of kin.

## Author Contributions

YY: wrote the paper. HJ, CWa, and NJ: collected patient data. SZ and CWu: summarized the data. QW and WJ: statistical analysis. ML, BD, XZ, and JP: modified paper. LW: consulted relevant literature. JZ and YZ: guide the project design. All authors contributed to the article and approved the submitted version.

## Conflict of Interest

The authors declare that the research was conducted in the absence of any commercial or financial relationships that could be construed as a potential conflict of interest.

## Publisher's Note

All claims expressed in this article are solely those of the authors and do not necessarily represent those of their affiliated organizations, or those of the publisher, the editors and the reviewers. Any product that may be evaluated in this article, or claim that may be made by its manufacturer, is not guaranteed or endorsed by the publisher.
